# Perceived vulnerability to COVID-19 infection from event attendance: results from Louisiana, USA, two weeks preceding the national emergency declaration

**DOI:** 10.1186/s12889-020-10035-6

**Published:** 2020-12-21

**Authors:** Ran Li, Bingcheng Yang, Jerrod Penn, Bailey Houghtaling, Juan Chen, Witoon Prinyawiwatkul, Brian E. Roe, Danyi Qi

**Affiliations:** 1 Department of Agricultural Economics and Agribusiness and the LSU AgCenter, 101 Martin D. Woodin Hall, Louisiana State University, Baton Rouge, Louisiana, 70803 USA; 2 School of Business, Sun Yat-sen University, 135 XinGangxi Road, Haizhu District, Guangzhou, 510275 China; 3 School of Nutrition and Food Sciences, 297 Knapp Hall, Louisiana State University and LSU AgCenter, Baton Rouge, Louisiana, 70803 USA; 4 Department of Human Resource Management, College of Humanities, Sichuan Agricultural University, 46 Xinkang Rd, Yucheng District, Ya’an, Sichuan China; 5 Department of Agricultural, Environmental and Development Economics, 2120 Fyffe Road, Ohio State University, Columbus, OH 43210 USA

**Keywords:** COVID-19, Infectious disease transmission, Social distancing, Risk reduction behavior, Guideline adherence, Louisiana, Higher education, College students, Classification tree analysis

## Abstract

**Background:**

Individual perceptions of personal and national threats posed by COVID-19 shaped initial response to the pandemic. The aim of this study was to investigate the changes in residents’ awareness about COVID-19 and to characterize those who were more aware and responsive during the early stages of the pandemic in Louisiana.

**Methods:**

In response to the mounting threat of COVID-19, we added questions to an ongoing food preference study held at Louisiana State University from March 3rd through March 12th, 2020. We asked how likely it was that the spread of the coronavirus will cause a national public health crisis and participants’ level of concern about contracting COVID-19 by attending campus events. We used regression and classification tree analysis to identify correlations between these responses and (a) national and local COVID case counts; (b) personal characteristics and (c) randomly assigned information treatments provided as part of the food preference study.

**Results:**

We found participants expressed a higher likelihood of an impending national crisis as the number of national and local confirmed cases increased. However, concerns about contracting COVID-19 by attending campus events rose more slowly in response to the increasing national and local confirmed case count. By the end of this study on March 12th, 2020 although 89% of participants agreed that COVID-19 would likely cause a public health crisis, only 65% of the participants expressed concerns about contracting COVID-19 from event attendance. These participants were significantly more likely to be younger students, in the highest income group, and to have participated in the study by responding to same-day, in-person flyer distribution.

**Conclusions:**

These results provide initial insights about the perceptions of the COVID-19 public health crisis during its early stages in Louisiana. We concluded with suggestions for universities and similar institutions as in-person activities resume in the absence of widespread vaccination.

**Supplementary Information:**

The online version contains supplementary material available at 10.1186/s12889-020-10035-6.

## Background

Individual perceptions of personal and national threats posed by COVID-19 have undoubtedly shaped initial public response to and ultimately the speed and geographical diffusion of the most disruptive public health crises in the past century [1]. Public perceptions are an important component of global responses to the pandemic. For example, public reaction to national level communications critically impacted how the pandemic unfolded in the United Kingdom [2]. Individual perception of infection risk is a critical parameter in epidemiological prediction models [3], but such perceptions may not be regularly collected despite their potential effect on individual and public health officials’ response times during critical action windows. For example, a single-day delay in COVID-19 response times across Chinese provinces during early 2020 significantly increased the newly confirmed case rate by 2.2%, which translated to an average of 497 more confirmed cases per 10,000 population per square kilometer [4]. Rapid response ultimately relies upon broad-based compliance by populations, which stems from the perceived risk of the evolving phenomenon among individuals.

Several empirical regularities in human response during epidemics provide context for understanding contemporary responses to COVID-19. For example, women have been found about 50% more likely to adopt non-pharmaceutical protective responses (e.g., mask wearing, hand washing) during several prior respiratory epidemics [5]. Among Dutch residents, those who were more likely to undertake preventative actions in response to the threats of Avian Influenza (AI) outbreaks (H5N1) were older, had less formal education, had obtained a flu vaccine, perceived higher severity of AI, perceived greater vulnerability to AI, and thought more about AI [6]. During the H1N1 influenza epidemic in Korea, female students reported higher perceptions of illness severity and of personal susceptibility to infection than men [7].

However, little is currently known about how individuals assess the national and personal risks associated with the COVID-19 pandemic during critical communication windows, including the early stages of disease spread prior to official government mandates. To our knowledge, and at the time of the study, the only published studies that examined perceptions of the COVID-19 threat came from surveys in China documenting demographic correlates of psychological impacts caused by the COVID crisis [8] and from online surveys in South Korea documenting psychological and behavioral responses during the early stages of COVID-19 transmission [9]. In China, women, students, and those reporting specific physical symptoms and unfavorable self-rated health reported significantly greater psychological impacts of COVID-19 [8]. In South Korea respondents who were female, older and perceived greater severity of COVID-19 were more likely to adopt precautionary behaviors and favored taking the behaviors they viewed as efficacious [9]. However, the United States is contextually different in terms of population and government response to the COVID-19 pandemic, which may lead to variations in patterns of response.

The aim of this article was to investigate individual responses to COVID-19 threats in the early stage of the pandemic using responses gathered in a study conducted during a period closely preceding the cancellation of in-person classes and events at Louisiana State University due to concerns about COVID-19. The study occurred at the University’s Baton Rouge campus in Louisiana, the state with the fastest reported growth rate of coronavirus cases in the world and the third highest number of cases per capita in the United States during the end of March 2020 [10].

## Methods

### Study design and setting

To understand individuals’ responses to the early outbreak of COVID-19, we asked study participants how likely it was that the spread of the coronavirus will cause a national public health crisis and their level of concern about contracting COVID-19 by attending campus events*.* Data were collected from participants in an ongoing study focused on understanding consumer food choice and consumption behavior during midday meals (11:00 AM – 2:00 PM). Students, staff, and faculty of Louisiana State University (LSU) were recruited to participate in a study held at the Food Sensory Services Lab on the Baton Rouge campus where they were offered a choice among several commercially prepared lunch options. They were provided a fixed budget for lunch and kept unspent budget as cash compensation. After providing informed consent, participants moved to isolated, individual kiosks with a computer to answer an online survey in which information treatments were randomly assigned and participants chose among a series of competing lunch options. One of the participant’s preferred lunch options was delivered by staff to the kiosk. Upon completing the meal, staff removed the food tray and the participant completed an online exit survey via the kiosk computer that focused on satisfaction with the provided meal and personal information.

Randomly assigned experimental elements included whether participants received information about food waste (vs. screen time, Food Waste Info); received information about improving nutrition (vs. financial literacy, Nutrition Info); received meals with more vegetables (vs. fewer, Vegetable Group); received meals on a large plate (vs. smaller, Large Plate); received meals on a compostable plate (vs. plastic, Compostable Plate); and received menus where the vegetable was listed at the top in the description of the offering (vs. lower, Veg Top of Menu). More detail and context concerning the experimental elements are included in the Supporting Information (Figure S2).

The food preference study initially began on February 17, 2020. In late February, as concerns about the spread of COVID-19 in the United States increased, we feared that the national emergence of COVID-19 could influence the composition of participants volunteering for this in-person study. To control for such potential changes, we added two questions to all exit surveys administered from March 3 to the final day of the study on March 12, 2020 (Figure S1): (1) In your opinion, how likely is it that the spread of COVID-19 (the coronavirus) will cause a public health crisis in the United States? (*National Likelihood*); and (2) How concerned are you that you will contract COVID-19 by attending events on campus (*Local Vulnerability*)? Responses to both questions were registered on a 5- point Likert scale (1 = very unlikely/unconcerned, 2 = moderately unlikely/unconcerned, 3 = neither likely/concerned nor unlikely/unconcerned, 4 = moderately likely/concerned, 5 = very likely/concerned). Responses to these two questions can provide insights into local perceptions of contemporaneously delivered state and federal COVID-19 communications critical for informing strategies to encourage public adherence to safety guidelines.

LSU continued all in-person classes and food service operations through March 13, 2020, and no official announcements were made regarding the cancellations of any on-campus activities before the end of the last study session (2: 00 PM March 12th) [11]. At 4:00 PM on March 12th, 2020, LSU’s official communications regarding COVID-19 first mentioned the cancellation of on-campus classes starting with the week of March 16th [12], and then announced the cancellation of non-class activities involving 30 people or more immediately at 11:30 AM on March 13, 2020 [13]. For reference, a national emergency was declared in response to COVID-19 the afternoon of March 13, 2020 [14].

### Participant recruitment and sampling

The sample used in this study included 356 participants enrolled from March 3rd through March 12th, 2020. Individuals were recruited via pre-existing email recruitment lists, flyers circulated on campus, advertising announcements in classes, and advertisements in university locations. Inclusion criteria included age 18 years or older with no dietary restrictions involving beef consumption. Three participants were omitted from all analyses because they failed to pass an attention test embedded in the survey, leaving an effective sample size of 353.

### Analysis

Descriptive statistics and regression analyses were conducted in Stata (version 16) and classification tree analyses were conducted using R (version 3.6.0). When more convenient for analysis, the 5-point Likert responses were simplified into binary variables (very or moderately likely/concerned = 1; all other responses = 0). We also defined the variable *National, not Local* to equal one when participants think a national crisis is very or moderately likely (*National Likelihood* > 3) but are neither very nor moderately concerned about contracting the virus by attending campus events (*Local Vulnerability* ≤ 3). Personal characteristics applied in the analyses included gender, age, student status (=1 if enrolled in University classes, =0 otherwise), household income, race, health insurance status, recycling frequency, experience with food composting, previous knowledge of food waste as an issue, whether they were trying to eat healthier, and whether they attended the session in response to in-person flyer distribution on the experiment date (as opposed to alternative recruitment such as emails or class announcements).

The timing of the study was included as a control variable in the model using several approaches. In one variant, we controlled for the number of confirmed cases nationwide in the United States as of 2:00 pm on the day of the study [15], while in another variant we controlled for the number of confirmed cases locally in Louisiana as of 2:00 pm [16–20]. A third variant, presented in the Supporting Information, controlled for the timing of the study with daily fixed effects (e.g., a separate variable denoting that the day of the study was March 3, March 4, etc.). In addition, personal characteristics were included in an ordered logit regression model of the Likert scale response to the two COVID-19 perception questions and in a binary logit regression model for the *National, not Local* variable. Statistical significance was set at the 5% level with results at the 10% level deemed marginally significant.

We also estimated classification trees for each dependent variable (the binary versions of *National Likelihood* and *Local Vulnerability*, plus *National, not Local*). Classification tree estimation is distinct from logit regressions in that they predict observations that are likely to be classified into a particular group (e.g., those for whom *National Likelihood* = 1) by splitting the sample into multiple sub-samples based on a series of predictor variables [21, 22]. The predictor variables included the explanatory variables used in the logit models. A version of classification tree analysis where the Gini improvement measure was used as the splitting criteria [23] was employed. A tree was grown first by splitting the sample based on the outcome of one of the predictor variables, and then branches were created to further subdivide the sample based on the levels of additional predictor variables. While classification tree analyses yield numerous outputs (see Supporting Information for standard graphical outputs), we focused on the relative variable importance scores, which measured the variable’s ability to predict the outcome in the estimated tree. Relative variable importance scores provided insights, for example, into which of several explanatory variables that are significant in a logit regression prove most critical for improving predictive capacity.

## Results

Descriptive statistics for key variables are presented in Table 1 with all other descriptive statistics provided in Supporting Information (Table S1).

**Table 1:** Sample Descriptive Statistics

VARIABLES	Mean or %
Dependent Variables:	
*National Likelihood* (Likert scale, 1–5)	3.89
*National Likelihood* (converted to binary)	0.74
*Local Vulnerability* (Likert scale, 1–5)	3.22
*Local Vulnerability* (converted to binary)	0.51
*National, not Local* (converted to binary)	0.29
Female	58.4%
Age × Student Status:	
18–24 × Non-student	24.3%
18–24 × Student	57.8%
25+ × Non-student	7.4%
25+ × Student	10.5%
Household income per year:	
Less than $15,000	17.3%
$15,000–$49,999	26.9%
$50,000 - $99,999	14.7%
$100,000 and above	14.5%
Prefer not to answer	26.6%
Race/Ethnicity	
Non – Hispanic White	52.7%
Non – Hispanic Black	21.5%
Other	25.8%
Hispanic or Latino	8.8%
Asian	12.5%
All other responses	4.5%
# of Observations	353

*Notes*: See supporting information for question wording and response options and for experimental element descriptions (Figure S1 and S2); and for descriptive statistics for the remaining explanatory variables (Table S1)

**Fig. 1:**
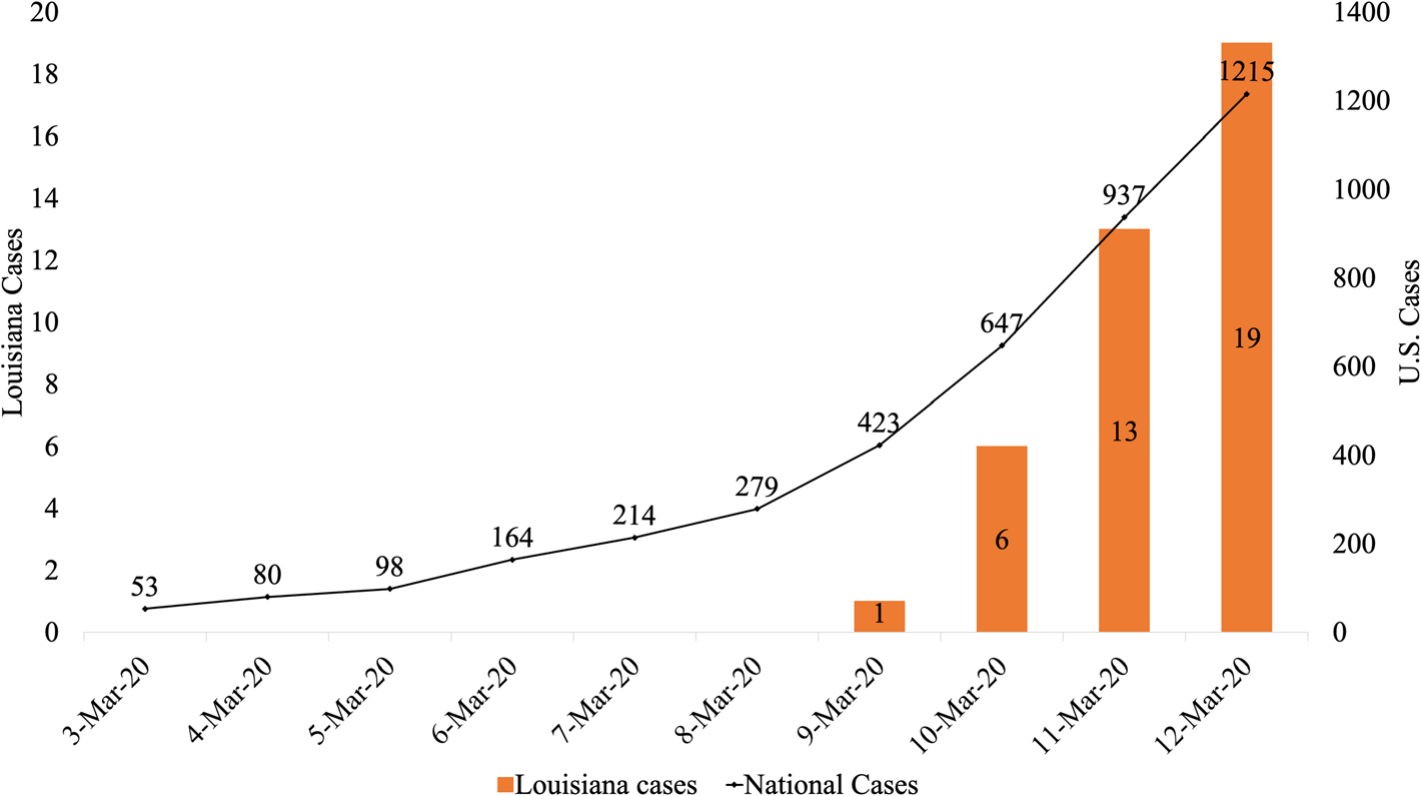
Confirmed Presumptive Positive COVID-19 Cases in the United States and Louisiana as Reported During the March 3–March 12, 2020 Study Period. *Information Source*: Centers for Disease Control and Prevention (CDC) [15], State of Louisiana: Office of the Governor [16, 19], Louisiana Department of Health [17, 18, 20]

**Fig. 2:**
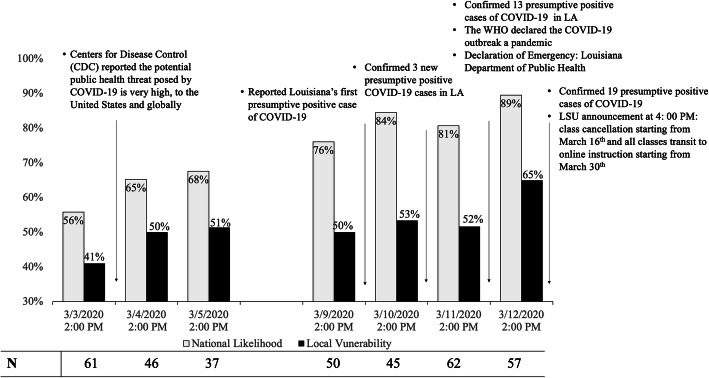
Responses to COVID-19 Questions by Study Day. *Note*: N represents the number of daily study participants. Public announcements occurred after daily study hours, which ended by 2 PM CST. The numbers presented in Fig. 2 and the data analysis presented below are based on CDC preliminary data available on the website at the time of analysis  *Information sources*: Centers for Disease Control and Prevention (CDC) [24], World Health Organization (WHO) [25], State of Louisiana: Office of the Governor [16, 19], Louisiana Department of Health [18, 20], LSU Coronavirus Updates & Information [11–13]

We used the number of presumptive positive COVID-19 cases reported nationally (line graph, right axis) and in Louisiana (bar graph, left axis) to characterize the progress of the pandemic during our study period in Fig. 1. Figure 2 traces the daily averages among study participants for the two COVID-19 questions, while highlighting key events in the evolution of COVID-19 timeline for Louisiana. Specifically, the gray bar depicts the percent who responded that COVID-19 was likely (moderately or very) to cause a national public health crisis while the black bars capture the percent that were concerned (moderately or very) that attendance at campus events would cause them to contract COVID-19.

Figure 1 shows the national case count (as reported during the study period) went from 53 on the first day of the study (March 3) to more than 1200 cases by the last day of the study (black line). Figure 2 juxtaposes the daily responses to the COVID-19 questions (grey and black bars) with key events in the national and Louisiana crisis timeline. No cases were identified and reported in Louisiana until the second week of the study (bars, Fig. 1) and LSU communications stated that no cases had been identified on campus [11]. However, a lack of testing in the United States and in Louisiana likely underrepresented the prevalence of COVID-19 at the time [24].

**Fig. 3:**
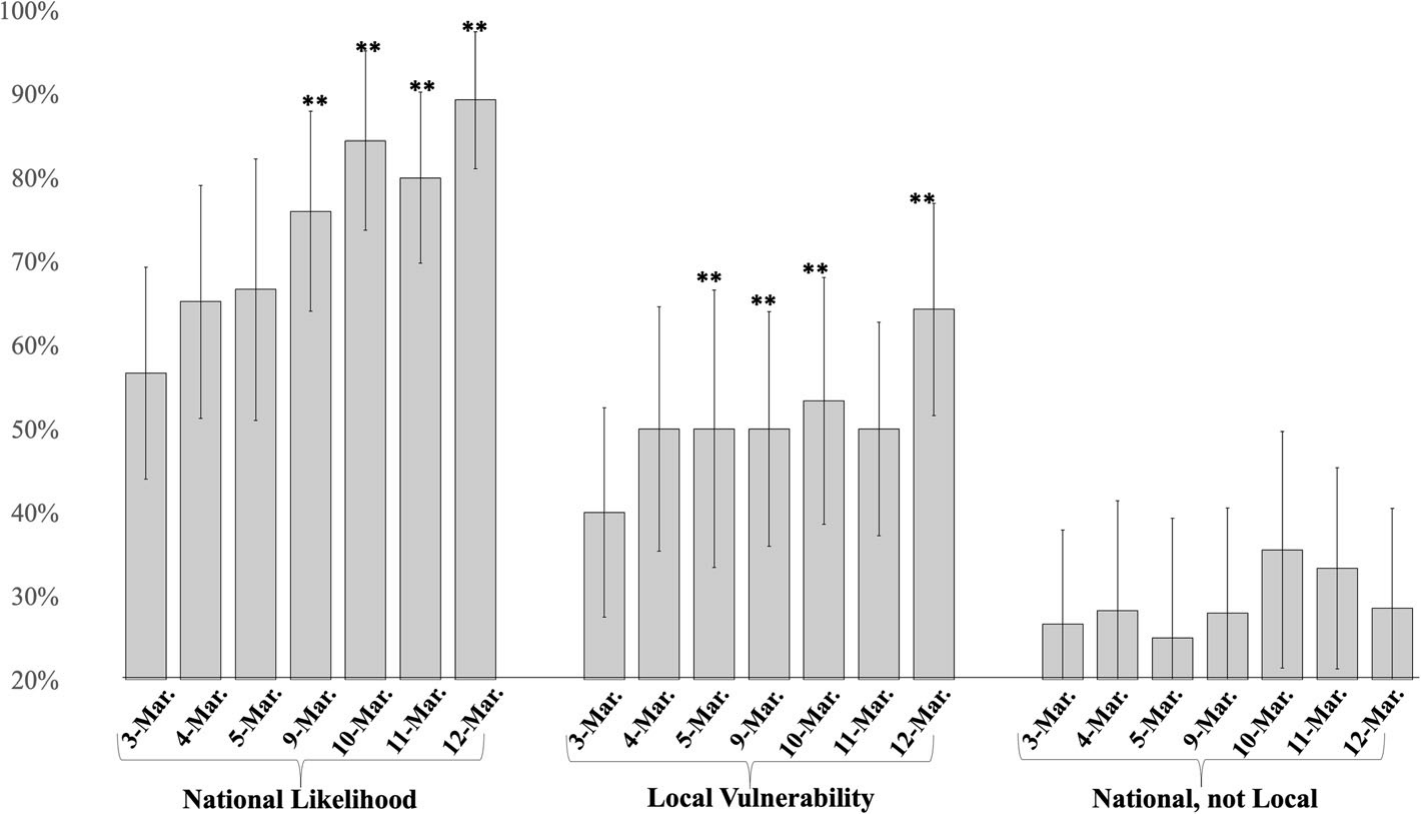
Daily Sample Means and 95% Confidence Intervals for Responses to COVID-19 Questions: (1) moderately or very likely that COVID-19 will cause a national public health crisis and (2) moderately or very concerned about contracting COVID-19 from attending campus events. The third group is the percent of participants who answered moderately/very likely to question (1) and did not answer moderately/very concerned to question (2). 95% confidence interval bars do not control for covariates. **, * denotes statistical difference between the value on this date and the value for the same variable on the first day of the study at the 5 and 10% level as determined by regression (Table S2) that controls for personal and experimental factors

Figure 3 shows the mean of *National Likelihood* (binary version), *Local Vulnerability* (binary version), and *National, not Local* over the experimental timeframe (bars, Fig. 3). We also noted the statistical difference of the value on each date from the value for the same variable on the first day of the study at the 5% and 10% level as determined by a regression (Table S2) that controlled for personal and experimental factors (stars, Fig. 3). *National Likelihood* increased steadily through the study period. Although, even on the final day of the study when the confirmed cases nationwide surpassed 1200, more than 10% of participants did not agree that a national crisis was likely. *National Likelihood* increased in an insignificant manner even though the confirmed cases nationwide almost doubled from March 3rd to March 5th, and *National Likelihood* was statistically greater than the first day of the study only from March 9 to March 12, i.e., the second week of the study period (Fig. 3: *National Likelihood*).

*Local Vulnerability* increased 10% points on March 4th, the day after the Centers for Disease Control and Prevention reported that COVID-19 was a potential global public health threat with the expectation for community spread in the United Sates [24]. *Local Vulnerability* remained relatively stable from March 4th to March 11th, a timeframe during which COVID-19 cases increased more than 10-fold across the United States and participants’ perceived *National Likelihood* increased about 20% points. *Local Vulnerability* featured significant increases compared to the first day of the study starting on March 5, the Friday of the first week of the study (Fig. 3: *Local Vulnerability*). The percent of participants in the *National, not Local* response pattern (agreeing a national crisis was likely but not expressing concern about attending campus events) stayed relatively constant over the entire period and featured no significant differences from the first day of the study (Fig. 3: *National, not Local*).

### Associations with COVID-19 question responses

Table 2 displays the estimated ordered logit results for *National Likelihood* and *Local Vulnerability* variables in their Likert scale form (1 = very unlikely/unconcerned, …*.,* 5 = very likely/concerned) and the estimated binary logit model for the *National, not Local* variable. The progress of the pandemic was measured and controlled in the model using two different approaches in Table 2. In columns (1) to (3), the number of confirmed cases reported in the United States before 2:00 PM on the experiment date was included as a control for the progress of the pandemic. In columns (4) to (6), the progress of the pandemic was measured by the reported confirmed cases in Louisiana.

**Table 2:** Regression models of COVID-19 question responses

	(1) Ordered Logit	(2) Ordered Logit	(3) Binary Logit	(4) Ordered Logit	(5) Ordered Logit	(6) Binary Logit
VARIABLES	*National Likelihood*	*Local Vulnerability*	*National, not Local*	*National Likelihood*	*Local Vulnerability*	*National, not Local*
**# of Cases confirmed**						
# of cases (100’s) in United States confirmed by 2:00 PM of the study date	0.173^b^	0.110^b^	−0.000			
	(0.038)	(0.036)	(0.045)			
# of cases in Louisiana confirmed by 2:00 PM of the study date				0.104^b^	0.073^b^	−0.004
				(0.026)	(0.025)	(0.031)
**Personal Characteristics:**						
Female	0.536^b^	0.099	0.178	0.563^b^	0.108	0.185
	(0.215)	(0.208)	(0.264)	(0.214)	(0.208)	(0.263)
Age × Student Status: (Base: 18–24 × Student)	*Joint p = 0.100*^a^	*Joint p = 0.027*^b^	*Joint p = 0.095*^a^	*Joint p = 0.093*^a^	*Joint p = 0.035*^b^	*Joint p = 0.091*^a^
25–44 × Student	0.273	0.858^b^	−0.366	0.198	0.803^b^	−0.370
	(0.381)	(0.371)	(0.486)	(0.380)	(0.370)	(0.485)
18–24 × Non-student	0.369	0.246	0.178	0.372	0.247	0.179
	(0.255)	(0.243)	(0.294)	(0.255)	(0.243)	(0.294)
25+ × Non-student	− 0.710^a^	0.965^b^	−1.773^b^	−0.750^a^	0.950^b^	−1.781^b^
	(0.430)	(0.412)	(0.773)	(0.429)	(0.411)	(0.772)
HH Income: (Base: $14,999 or less per year)	*Joint p = 0.118*	*Joint p = 0.258*	*Joint p = 0.336*	*Joint p = 0.082*^a^	*Joint p = 0.218*	*Joint p = 0.338*
$15,000–$49,999 per year	0.081	−0.453	0.369	0.053	− 0.468	0.369
	(0.330)	(0.316)	(0.406)	(0.330)	(0.316)	(0.406)
$50,000 - $99,999 per year	−0.668^a^	− 0.230	− 0.109	− 0.719^a^	−0.270	− 0.106
	(0.372)	(0.361)	(0.460)	(0.372)	(0.362)	(0.460)
$100,000 or more per year	−0.400	− 0.766^b^	0.716	− 0.428	− 0.801^b^	0.717
	(0.370)	(0.352)	(0.438)	(0.371)	(0.352)	(0.438)
Prefer not to answer	−0.566^a^	− 0.433	0.107	− 0.627^b^	− 0.485	0.107
	(0.318)	(0.308)	(0.394)	(0.318)	(0.309)	(0.394)
Race: (Base: Non- Hispanic White)	*Joint p = 0.500*	*Joint p = 0.011*^b^	*Joint p = 0.221*	*Joint p = 0.503*	*Joint p = 0.015*^b^	*Joint p = 0.213*
Non-Hispanic Black	0.213	0.590^b^	−0.573	0.171	0.564^b^	−0.577^a^
	(0.292)	(0.278)	(0.349)	(0.291)	(0.277)	(0.348)
All Others	−0.156	0.698^b^	−0.348	− 0.190	0.672^b^	− 0.351
	(0.261)	(0.256)	(0.333)	(0.260)	(0.255)	(0.331)
Health Insurance	−0.118	0.202	0.196	−0.151	0.177	0.195
	(0.339)	(0.325)	(0.429)	(0.336)	(0.324)	(0.428)
Recycle	0.543	0.624^a^	0.097	0.570	0.643^a^	0.099
	(0.374)	(0.328)	(0.419)	(0.376)	(0.329)	(0.419)
Compost	0.071	0.210	−0.108	0.010	0.184	−0.111
	(0.233)	(0.227)	(0.286)	(0.231)	(0.226)	(0.285)
Aware of Food Waste	−0.121	0.039	−0.018	− 0.110	0.051	− 0.019
	(0.215)	(0.209)	(0.261)	(0.215)	(0.209)	(0.261)
Eat a Healthy Diet	−0.547^b^	− 0.285	− 0.004	−0.553^b^	− 0.297	−0.003
	(0.269)	(0.256)	(0.331)	(0.269)	(0.257)	(0.331)
In-Person Recruitment	0.135	−0.188	0.698^b^	0.143	−0.185	0.697^b^
	(0.228)	(0.214)	(0.272)	(0.229)	(0.215)	(0.272)
**Randomly Assigned Experimental Elements:**						
Food Waste Info	0.378^a^	0.550^b^	−0.223	0.371^a^	0.550^b^	−0.224
	(0.206)	(0.201)	(0.252)	(0.206)	(0.201)	(0.252)
Nutrition Info	0.191	−0.072	0.314	0.183	−0.077	0.313
	(0.206)	(0.200)	(0.250)	(0.206)	(0.200)	(0.250)
Vegetable Group	−0.424^a^	−0.001	−0.069	− 0.383	−0.009	− 0.050
	(0.246)	(0.234)	(0.293)	(0.248)	(0.237)	(0.297)
Large Plate	0.340	−0.067	0.232	0.339	−0.050	0.225
	(0.223)	(0.214)	(0.270)	(0.224)	(0.215)	(0.271)
Compostable Plate	0.189	0.034	0.051	−0.045	−0.119	0.055
	(0.224)	(0.214)	(0.267)	(0.222)	(0.213)	(0.265)
Veg Top of Menu	0.046	−0.187	0.247	0.043	−0.185	0.246
	(0.213)	(0.204)	(0.257)	(0.213)	(0.204)	(0.257)
Constant cut1 Constant cut2	−2.342^b^	−0.932		−2.799^b^	−1.232^b^	
	(0.691)	(0.627)		(0.682)	(0.611)	
	−0.803	0.614		−1.270^b^	0.314	
	(0.657)	(0.621)		(0.647)	(0.604)	
Constant cut3	−0.031	1.173^a^		−0.506	0.872	
	(0.655)	(0.623)		(0.643)	(0.605)	
Constant cut4	1.940^b^	2.823^b^		1.444^b^	2.519^b^	
	(0.662)	(0.640)		(0.646)	(0.620)	
Constant			−1.753^b^			− 1.744^b^
			(0.779)			(0.760)
Observations	353	353	353	353	353	353
Pseudo R^2^	0.063	0.046	0.071	0.058	0.045	0.071

*Notes*: ^b^, ^a^ denotes a statistical significance at the 5 and 10% level. Standard errors of the estimated logit coefficient parameters are presented in parentheses. Variable importance factors from complementary Classification Tree Analyses (see Table 3 and Figures S3-S4 for full classification tree results), where larger numbers represent variables with greater predictive capacity

Both *National Likelihood* and *Local Vulnerability* increased significantly as the national and local confirmed cases increased (Table 2). Furthermore, the relative variable importance scores from the classification tree results (Table 3), which rank variables by their ability to improve the classification tree’s predictive capacity, confirm the important role that the national and Louisiana case count play in understanding responses to these two survey questions. Note, the classification tree analysis for *National Likelihood* resulted in a single node, and hence these results were omitted from Table 3. For *Local Vulnerability*, the classification tree suggests that the number of national cases was closely followed by the number of Louisiana cases in terms of importance for accurate prediction.

**Table 3:** Relative Importance Scores for Predictor Variables from Classification Tree Analyses by Outcome Variable

*Local Vulnerability*	Score	*National, not Local*	Score
National Cases	100.00	*National Likelihood*	100.00
LA Cases	95.96	Income > 100 k	21.33
Compostable Plate	59.92	Nutrition Info	17.26
Recycle: Yes	44.62	LA Cases	14.09
Food Waste Info	41.90	National Cases	14.09
Nutrition Info	41.24	In-Person Recruitment	12.34
Race: Non-Hispanic White	39.76	Aware of Food Waste	9.67
Vegetable Group	25.30	Income: $50 k-$100 k	6.04
Race: Non-Hispanic Black	23.37	Vegetable Group	6.04
Income: $15 k - $50 k	14.05	Race: Non-Hispanic Black	5.27
18–24 x Student	10.45	18–24 x Student	3.11
Large Plate	10.18	Recycle: Yes	2.84
Income > 100 k	7.45	Race: Non-Hispanic White	2.66
In-Person Recruitment	5.89	Compost: Yes	1.66
Female	4.07	25–44 x Student	1.66
Compost: Yes	2.95	Income < 15 k	1.49
Eat a Healthy Diet	2.91	18–24 x Non-student	0.74
18–24 x Non-student	1.82	Large plate	0.74
FW heard	1.02	Female	0.23

*Note*: Higher scores indicate variables that were more pivotal in predicting the italicized outcome variables. See additional classification tree outputs in Figures S3-S4

Several personal characteristics were significantly associated with *National Likelihood* (column (1) and (4)). Men and those trying to eat a healthier diet provided significantly lower likelihood ratings. Staff or faculty who are older than 25 years old and those who earn $50,000–99,999 household income per year or prefer not to report their income provided marginally lower likelihood ratings, while participants who were randomly given the food waste information treatment as part of the experimental design provided marginally higher likelihood ratings than participants provided the screen time information treatment (Table 2).

Personal characteristics that were significantly positively associated with *Local Vulnerability* (column (2) and (5)) included being 25 years or older (regardless of student status) and identifying with a race other than non-Hispanic white. Those in the highest income category ($100,000 or more) displayed significantly lower *Local Vulnerability* than those earning less than $15,000 per year. In terms of randomly assigned elements from the food study’s experimental design, the only significant variable in the logit regression involved participants that received the food waste information treatment (rather than the screen time information treatment); this group reported significantly higher *Local Vulnerability*. Two marginally significant variables arised in the logit regression model of *National Likelihood*: participants who received food waste information provided marginally higher ratings while participants who received menu options with a larger proportion of vegetables provided marginally lower ratings (Table 2).

The classification tree analysis for *Local Vulnerability* implicated several variables as relatively important predictor variables that did not register as significant in the ordered logit regression model. This included participants randomly assigned compostable (rather than plastic) plates; those who reported past recycling behavior; and those who received information about the importance of nutrition (rather than information about the importance of financial literacy). The probability of a participant being classified as *National, not Local* in the logit regression model was not significantly associated with the number of confirmed cases nationwide or locally (Table 2), although the classification tree analysis revealed both the national and Louisiana case count as tied for the fourth most pivotal variable for prediction (Table 3).

Only two variables were significant in the *National, not Local* regression model (column (3) and (6)). Older (≥25 years), non-students were less likely to feature this response pattern than younger students while those who attended the experiment in response to in-person flyers were more likely to feature this response pattern (Table 2). The classification tree analysis found income to be a pivotal prediction variable, with those reporting household income greater than $100,000 more likely to be in a group with a high percent of participants with *National, not Local* = 1 (Table 3).

No experimental treatments were significant in the logit regression for *National, not Local* (Table 2). In the classification tree analysis, the provision of nutrition information (rather than financial literacy information) was a pivotal variable as those receiving this information more likely to be in groups with a higher percent having *National, not Local* = 1 (Table 3).

## Discussion

The spread of epidemics can be dramatically delayed or mitigated if individual perception of the risk of the epidemic is sufficiently large and leads to reduced community contact [26]. For example, early public warnings about the epidemic could improve population awareness and response to the national public crisis and hence lead to dramatic reductions in peak prevalence and size of the infected population. Such a strategy is highly sensitive to the translation of risk perception to self-prophylaxis measures, such as mask-wearing and social distancing, which slows community spread. While we did not elicit explicit measures of such behaviors, we did assess participants’ perceived vulnerability to contracting COVID-19 from attending campus events (*Local Vulnerability*), which may signal a willingness to undertake social distancing and other beneficial behaviors and be a behavioral precursor.

The first insight from observing the raw data plot in Fig. 2 is that *Local Vulnerability* persistently lags *National Likelihood*, and does not exceed the 50% mark until the last day of the study, which is the first day after the state of Louisiana had declared a public health emergency but before LSU had cancelled classes or campus events. Regression and classification tree analyses revealed some personal characteristics significantly associated with *National Likelihood* that align with the previous literature, e.g., women perceive a national public health crisis as more likely than men [27, 28]. In addition, participants younger than 25 and those identifying as non-Hispanic white are less likely to express *Local Vulnerability*, while those in the highest income category expressed lower *Local Vulnerability* than those in the lowest income bracket (Table 2). These results largely align with other findings from the general literature on risk perception. For example, one investigation found drivers aged 25 and older perceived significantly higher risk from aggressive driving tactics than younger drivers [29], and another found that respondents with higher income expressed less concern about environmental risks, which may stem from a heightened sense of material risk faced by those with lower incomes [30]. Race/ethnicity has also been linked with risk perceptions, for example non-Hispanic white, (particularly men), registered significantly lower environmental risk perceptions [31], suggesting that socio-political factors including power and status may influence risk perceptions.

We also identified several significant associations with personal characteristics that have no precedent in the extant literature, e.g., participants who were trying to eat a healthier diet were significantly negatively associated with *National Likelihood* (from the logit regression, Table 2), and those who reported any level of past recycling behavior were more likely to express concern about getting COVID-19 from attending campus events (classification tree analyses, Table 3 and Figure S3). Several randomly assigned information treatments presented as part of the food preference study also yielded statistically significant (logit regression) or pivotal (classification tree) relationships. Participants who received information about the social and financial costs of food waste were significantly more likely to express *Local Vulnerability* and marginally more likely to report higher *National Likelihood* ratings in the logit regression results (Table 2). The classification tree analyses identified that participants who received compostable paper plates were more likely to express high *Local Vulnerability* and that those who received nutrition information were more likely to have *National, not Local* = 1 (Table 3, Figures S3- S4).

While we cannot provide a definitive explanation of these novel relationships given post-hoc design constraints, we noted the food waste information treatment emphasized national-level and household-level implications of an individual behavior (e.g., food waste causing $161 billion of losses at the national level and $1500 of losses in an average household). Participants who linked the implications of individual behaviors to issues of sustainability may reflect more critically on the implications of personal actions during a public health crisis, which could help increase compliance with social distancing and other preventative behaviors. Such a pathway might also comport with the finding concerning recycling, where past recycling may have been an outcome of participants translating concerns about related public issues (e.g., environmental and resource degradation) into personal action (recycling behavior). Further, those who are overly concerned about healthy diets (or who are prompted to think about healthy diets) may be (or become) more inwardly directed, which distracts their attention from things that are outside themselves, e.g., a public health crisis like COVID-19. However, future research is required to understand if such clustering and crowding-out effects of individual protective attitudes and behaviors are robust findings, and what mediating factors are at play.

The results from this study could also reflect a more nuanced relationship between dietary and health aspirations and national public health perceptions. For example, dissemination of nutrition misinformation through media channels is long-standing problem in the U.S. [32], and further evidence of this has emerged specific to the COVID-19 pandemic [33]. It is possible that those aspiring to eat healthy diets could perceive less susceptibility to infectious disease based on salient, popular information that commonly describes nutrition evidence out of context or overstates implications of healthful eating for health and well-being, though more research is required to test such hypotheses.

We also found a persistent group of about 30% of participants who, for the entire study period regardless of the increase of the confirmed cases nationwide and locally, do not translate their perceived likelihood of a national public health crisis into personal vulnerability from attending campus events (*National, not Local*). These are likely a critical group in terms of modeling diffusion of COVID-19, as Poletti and colleagues [26] emphasized the role of translating perceived risk into preventative behaviors such as social distancing. Logit regression provided few insights into the characteristics associated with the *National, not Local* group other than older, non-students were less likely to feature this response pattern and those who spontaneously attended the study in response to same-day receipt of flyers were more likely. The classification tree results suggested that not all participants who responded to in-person recruitment were equally likely to be classified into *National, not Local* group. Those who received information about healthy eating (rather than financial literacy) information treatment were more likely to fall into this important group, while for certain branches of the classification tree, those participants in the highest reported income category are much more likely to be in the *National, not Local* group (see Figure S4).

We noted that the study was originally designed to investigate a topic other than COVID-19 perceptions, hence logical experimental treatments and additional questions about personal perceptions and behaviors relevant to understanding and predicting the spread of COVID-19 were not included and the questions that were posed were not motivated by theory. Another study limitation is that the sample was drawn from a single academic institution, limiting the representativeness of the data geographically, demographically, and socioeconomically. Finally, the data were acquired prior to the declaration of a national emergency, and we would expect further evolution in how people in this location might respond to these questions in the face of more dire national promulgations concerning the pandemic.

## Conclusions

Understanding perceptions related to risk can help to tailor national or local responses to curb transmission of infectious disease. While some characteristics that were significantly associated with a lower perceived local vulnerability to contracting COVID-19 have precedent from previous risk perception research (e.g., younger than 25, non-Hispanic white, higher income), others are novel and suggest the need for more investigation (e.g., relationship between self-reported healthy eating and infectious disease risk). Also, our finding that participants who were randomly assigned an information treatment that emphasized the national and household implications of food waste expressed significantly higher perceptions of local vulnerability may suggest that information campaigns emphasizing the national and household implications of individual behaviors could help increase compliance with social distancing and other preventative behaviors.

Throughout the study period, including the day after the emergency declaration, and about 30% of participants did not convert national perceptions of a likely public health crisis into perceived vulnerability from local event attendance. This could be a key group to target as localities and states strive to maintain social distancing policies and procedures or are in preparation for directives that may be needed should localities face additional spikes in infection rates. The significant characteristics associated with this group are limited, but do include age, with students less than 25 years of age more likely to fall into this group than older, non-students. This suggests potential challenges for universities as they integrate students back into campus life even as many epidemiological models suggest another wave of infections may arise. This provides evidence consistent with strategies that tailor communications efforts to younger cohorts that encourage social distancing, mask-wearing and other prevention behaviors by emphasizing both public and personal implications of failure to adhere to these practices.

## Supplementary Information

**Additional file 1: Table S1.** Descriptive statistics not included in Table 1; **Table S2.** Regression results establishing significance in Fig. 3; **Figure S1.** Exit survey. **Figure S2.** Experimental treatment details; **Figure S3.** Classification tree results for Local Vulnerability; **Figure S4.** Classification tree results for National not Local.

**Additional file 2.** Data set used in analysis.

## Data Availability

All data analyzed during this study are included in the supplementary information files.

## Abbreviations

COVID-19: Corona virus disease 2019
WHO: World Health Organization

## References

[1] Gates B (2020). Responding to Covid-19—a once-in-a-century pandemic?. N Engl J Med.

[2] Cowper A. Covid-19: are we getting the communications right? BMJ. 2020;368:m919. 10.1136/bmj.m919 32144115

[3] Bagnoli F, Lio P, Sguanci L (2007). Risk perception in epidemic modeling. Phys Rev E.

[4] Zhang Q, Deng H, Zhang C. The value of early response by surrounding areas of epidemic center Hubei during COVID-2019 outbreak in China: A quasi-experiment analysis. Available at SSRN 3548372 2020.

[5] Moran KR, Del Valle SY. A meta-analysis of the association between gender and protective behaviors in response to respiratory epidemics and pandemics. PLoS ONE. 2016;11(10):e0164541. 10.1371/journal.pone.0164541. 10.1371/journal.pone.0164541 PMC5074573 27768704

[6] de Zwart O, Veldhuijzen IK, Richardus JH, Brug J (2010). Monitoring of risk perceptions and correlates of precautionary behaviour related to human avian influenza during 2006-2007 in the Netherlands: results of seven consecutive surveys. BMC Infect Dis.

[7] Park JH, Cheong HK, Son DY, Kim SU, Ha CM (2010). Perceptions and behaviors related to hand hygiene for the prevention of H1N1 influenza transmission among Korean university students during the peak pandemic period. BMC Infect Dis.

[8] Wang C, Pan R, Wan X, Tan Y, Xu L, Ho CS, Ho RC (2020). Immediate psychological responses and associated factors during the initial stage of the 2019 coronavirus disease (COVID-19) epidemic among the general population in China. Int J Environ Res Public Health.

[9] Lee M, You M (2020). Psychological and behavioral responses in South Korea during the early stages of coronavirus disease 2019 (COVID-19). Int J Environ Res Public Health.

[10] Silverman H. Louisiana governor says his state has the fastest growth rate of coronavirus cases in the world. 2020. https://www.cnn.com/2020/03/23/us/louisiana-coronavirus-fastest-growth/index.html. Accessed 10 May 2020.

[11] Louisiana State University. Message regarding coronavirus. 2020. https://www.lsu.edu/coronavirus/messages/email/2020-03-11-coronavirus.php. Accessed 9 May 2020.

[12] Louisiana State University. Coronavirus update. 2020. https://www.lsu.edu/coronavirus/messages/email/2020-03-12-update.php. Accessed 9 May 2020.

[13] Louisiana State University. LSU event canceled immediately. 2020. https://www.lsu.edu/coronavirus/messages/email/03-13-events-canceled.php. Accessed 9 May 2020.

[14] Trump DJ. Proclamation on declaring a national emergency concerning the novel coronavirus disease (COVID-19) outbreak. 2020. https://www.whitehouse.gov/presidential-actions/proclamation-declaring-national-emergency-concerning-novel-coronavirus-disease-covid-19-outbreak/. Accessed 9 May 2020.

[15] Centers for Disease Control and Prevention. Number of COVID-19 Cases in the U.S., by Date Reported. 2020. https://www.cdc.gov/coronavirus/2019-ncov/cases-updates/previouscases.html. Accessed 28 Mar 2020.

[16] State of Louisiana: Office of Governor. Reported Louisiana’s first presumptive positive case of COVID-19. 2020. https://gov.louisiana.gov/index.cfm/newsroom/detail/2392. Accessed 9 May 2020.

[17] Louisiana Department of Health. Health officials confirms two new presumptive positive COVID-19 cases in Louisiana as state increases testing for the virus. 2020. http://ldh.la.gov/index.cfm/newsroom/detail/5463. Accessed 9 May 2020.

[18] Louisiana Department of Health. Health officials confirm three new presumptive positive COVID-19 cases in Louisiana as state increases testing for the virus. 2020. http://ldh.la.gov/index.cfm/newsroom/detail/5464. Accessed 9 May 2020.

[19] State of Louisiana: Office of Governor. Gov. Edwards declares public health emergency in response to COVID-19. 2020. https://gov.louisiana.gov/index.cfm/newsroom/detail/2400. Accessed 9 May 2020.

[20] Louisiana Department of Health. As of 4:45 p.m. March 12, 2020, the Louisiana Department of Health is reporting 19 presumptive positive cases of #COVID19 in Louisiana. Louisiana Department of Health. 2020. https://twitter.com/LADeptHealth/status/1238228144483643394. Accessed 9 May 2020.

[21] Varian HR (2014). Big data: new tricks for econometrics. J Econ Perspect.

[22] Lusk JL (2017). Consumer research with big data: applications from the food demand survey (FooDS). Am J Agric Econ.

[23] Lemon SC, Roy J, Clark MA, Friedmann PD, Rakowski W (2003). Classification and regression tree analysis in public health: methodological review and comparison with logistic regression. Ann Behav Med.

[24] Centers for Disease Control and Prevention. Coronavirus disease 2019 (COVID-19) situation summary. 2020. https://www.cdc.gov/coronavirus/2019-ncov/cases-updates/summary.html. Accessed 9 May 2020.

[25] World Health Organization. WHO characterizes COVID-19 as a pandemic. World Health Organization. 2020. https://www.who.int/emergencies/diseases/novel-coronavirus-2019/events-as-they-happen. Accessed 13 Dec 2020.

[26] Poletti P, Ajelli M, Merler S (2012). Risk perception and effectiveness of uncoordinated behavioral responses in an emerging epidemic. Math Biosci.

[27] Rolison JJ, Shenton J (2020). How much risk can you stomach? Individual differences in the tolerance of perceived risk across gender and risk domain. J Behav Decis Mak.

[28] Silver ER, Hur C (2020). Gender differences in prescription opioid use and misuse: implications for men's health and the opioid epidemic. Prev Med.

[29] Rhodes N, Pivik K (2011). Age and gender differences in risky driving: the roles of positive affect and risk perception. Accid Anal Prev.

[30] Lo AY (2014). Negative income effect on perception of long-term environmental risk. Ecol Econ.

[31] Flynn J, Slovic P, Mertz CK (1994). Gender, race, and perception of environmental health risks. Risk Anal.

[32] Ayoob KT, Duyff RL, Quagliani D (2002). Position of the American dietetic association: food and nutrition misinformation. J Am Diet Assoc.

[33] U.S. Federal Trade Commission. FTC, FDA send warning letters to seven companies about unsupported claims that products can treat or prevent coronavirus: Commission continues efforts to protect consumers from deceptive advertising. 2020. https://www.ftc.gov/news-events/press-releases/2020/03/ftc-fda-send-warning-letters-seven-companies-about-unsupported. Accessed 9 May 2020.

