# Supplementary material for: Perceived vulnerability to COVID-19 infection from event attendance: results from Louisiana, USA, two weeks preceding the national emergency declaration
Source: BMC Public Health. 2020 Dec 21;20:1922. doi: 10.1186/s12889-020-10035-6 (PMC7750394; doi:10.1186/s12889-020-10035-6)
Supplement: Supplementary file 1 — Additional file 1: Table S1. Descriptive statistics not included in Table 1; Table S2. Regression results establishing significance in Fig. 3; Figure S1. Exit survey. Figure S2. Experimental treatment details; Figure S3. Classification tree results for Local Vulnerability; Figure S4. Classification tree results for National not Local. [file 12889_2020_10035_MOESM1_ESM.docx]

**Supporting Information**

Table S1. Descriptive statistics not included in Table 1

Table S2. Regression results establishing significance in Fig 3

Figure S1. Exit survey

Figure S2. Experimental treatment details

Figure S3. Classification tree results for *Local Vulnerability*

Figure S4. Classification tree results for *National, not Local*

**Table S1. Descriptive statistics not included in Table 1**

| VARIABLES | Mean or % |
| --- | --- |
| Health insurance = yes | 88.1% |
| Recycle (sometime, about ½ the time, most of the time, or whenever possible) | 89.2% |
| Ever lived in a household that composts food | 28.6% |
| Heard about food waste | 46.7% |
| Attempt to eat a healthy diet (agree) | 80.2% |
| In-person recruitment | 40.2% |
|  |  |
| Randomly Assigned Experimental Elements |  |
| Food Waste Info | 50% |
| Nutrition Info | 50% |
| Vegetable Group | 32% |
| Large Plate | 63% |
| Compostable Plate | 49% |
| Veg Top of Menu | 45% |
|  |  |
| Study Date |  |
| Mar 3^rd^ | 17.0% |
| Mar 4^th^ | 13.0% |
| Mar 5^th^ | 10.2% |
| Mar 9^th^ | 14.2% |
| Mar 10^th^ | 12.8% |
| Mar 11^th^ | 17.0% |
| Mar 12^th^ | 15.9% |
|  |  |
| # of Observations | 353 |

**Table S2. Regression models of COVID-19 question responses used in Fig 3**

|  | (1) Ordered Logit | (2) Ordered Logit | (3) Binary Logit |
| --- | --- | --- | --- |
| VARIABLES | *National Likelihood* | *Local Vulnerability* | *National Likelihood, Not Local Vulnerability* |
| **Personal Characteristics:** |  |  |  |
|  |  |  |  |
| Female | 0.531** | 0.092 | 0.195 |
|  | (0.218) | (0.212) | (0.267) |
|  |  |  |  |
| Age × Education: (Base: 18-24 × Student) | *Joint p=0.097** | *Joint p=0.016*** | *Joint p=0.096** |
| 25-44 × Student | 0.351 | 0.920** | -0.365 |
|  | (0.384) | (0.377) | (0.493) |
| 18-24 × Non-student | 0.401 | 0.302 | 0.168 |
|  | (0.259) | (0.247) | (0.297) |
| 25+ × Non-student | -0.643 | 1.047** | -1.803** |
|  | (0.433) | (0.419) | (0.779) |
|  |  |  |  |
| HH Income: (Base: < $15,000 or less per year) | *Joint p=0.122* | *Joint p=0.249* | *Joint p=0.323* |
| $15,000-$49,999 per year | 0.099 | -0.387 | 0.325 |
|  | (0.339) | (0.322) | (0.412) |
| $50,000 - $99,999 per year | -0.684* | -0.294 | -0.131 |
|  | (0.381) | (0.370) | (0.470) |
| $100,000 or more per year | -0.435 | -0.814** | 0.728* |
|  | (0.375) | (0.357) | (0.442) |
| Prefer not to answer | -0.535 | -0.431 | 0.090 |
|  | (0.327) | (0.316) | (0.398) |
|  |  |  |  |
| Race: (Base: White) | *Joint p=0.651* | *Joint p=0.029*** | *Joint p=0.240* |
| Black | 0.142 | 0.428 | -0.586 |
|  | (0.300) | (0.286) | (0.359) |
| Others | -0.153 | 0.670** | -0.308 |
|  | (0.263) | (0.260) | (0.336) |
| Health Insurance | -0.073 | 0.231 | 0.196 |
|  | (0.340) | (0.324) | (0.432) |
| Recycle | 0.443 | 0.482 | 0.107 |
|  | (0.380) | (0.334) | (0.429) |
| Compost | 0.072 | 0.159 | -0.074 |
|  | (0.236) | (0.229) | (0.289) |
| Aware of Food Waste | -0.141 | 0.023 | 0.005 |
|  | (0.218) | (0.211) | (0.263) |
| Eat a Healthy Diet | -0.591** | -0.369 | -0.005 |
|  | (0.273) | (0.262) | (0.337) |
| In-Person Recruitment | 0.107 | -0.225 | 0.732** |
|  | (0.231) | (0.217) | (0.276) |
| **Randomly Assigned Experimental Elements:** |  |  |  |
|  |  |  |  |
| Food Waste Info | 0.373* | 0.575** | -0.214 |
|  | (0.206) | (0.202) | (0.253) |
| Nutrition Info | 0.188 | -0.113 | 0.315 |
|  | (0.207) | (0.201) | (0.251) |
| Vegetable Group | -0.263 | 0.206 | -0.522 |
|  | (0.421) | (0.397) | (0.499) |
| Large Plate | 0.434 | -0.005 | 0.170 |
|  | (0.342) | (0.330) | (0.408) |
| Compostable Plate | 0.723* | 0.702* | -0.279 |
|  | (0.404) | (0.388) | (0.479) |
| Veg Top of Menu | 0.079 | -0.120 | 0.241 |
|  | (0.215) | (0.205) | (0.260) |
|  |  |  |  |
| **Study Date:** (Base: March 3^rd^) | *Joint p=0.001*** | *Joint p=0.006*** | *Joint p=0.933* |
| Mar 4^th^ | 0.160 | 0.307 | 0.606 |
|  | (0.572) | (0.548) | (0.694) |
| Mar 5^th^ | 1.082 | 1.520** | -0.509 |
|  | (0.712) | (0.674) | (0.857) |
| Mar 9^th^ | 1.246** | 1.256** | -0.448 |
|  | (0.613) | (0.582) | (0.724) |
| Mar 10^th^ | 1.672** | 1.608** | -0.145 |
|  | (0.567) | (0.539) | (0.665) |
| Mar 11^th^ | 1.467** | 0.862 | 0.347 |
|  | (0.607) | (0.578) | (0.711) |
| Mar 12^th^ | 1.870** | 1.628** | 0.118 |
|  | (0.545) | (0.513) | (0.620) |
| Constant |  |  | -1.435 |
|  |  |  | (0.995) |
| Observations | 353 | 353 | 353 |
| Pseudo R^2^ | 0.067 | 0.054 | 0.076 |

**Figure S1. Exit survey.**

In the last 12 months, have you read, seen or heard anything about the amount of food that is wasted or about ways to reduce the amount of food that is wasted?

-Yes

-Maybe

-No

To what extent you agree with the following statements, in the last month: I attempt to eat a healthy diet.

-Strongly agree

-Somewhat agree

-Neither agree nor disagree

-Somewhat disagree

-Strongly disagree

What is your gender?

-Male

-Female

-Prefer not to answer

What is your age (in years)?

-18-24

-25-44

-45-64

-65+

What race would you consider yourself? (Please select only one answer)

-White non-Hispanic

-Black/African-American non-Hispanic

-Hispanic or Latino

-Asian

-Other

- Prefer not to answer

What is your employment status?

Employed full-time

Employed part time

Retired

Student

Other

Prefer not to answer

What is your classification? [Asked if employment status = ‘Student’]

-Freshman

-Sophomore

-Junior

-Senior

-Master's

-Doctoral

-Law/Veterinary Medicine

-Prefer not to answer

What is the highest level of education you have completed? [Asked if employment status is something other than ‘Student’]

-Middle school or less

-Some high school

-High school diploma/GED

-Some college/2-year degree

-Bachelor's degree

-Postgraduate degree

-Prefer not to answer

What is the total income for your household before taxes during the past 12 months?

-Less than $14,999 per year

-$15,000-$24,999 per year

-$25,000 - $49,999 per year

-$50,000 - $99,999 per year

-$100,000 and above per year

-Prefer not to answer

Do you have any health insurance?

-Yes

-No

-Prefer not to answer

About how often do you recycle cans, bottles, and paper?

-Whenever possible

-Most of the time

-About half the time

-Sometime

-Never

-Prefer not to answer

Have you ever lived in a household where uneaten food was composted?

-Yes

-No

-Unsure

In your opinion, how likely is it that the spread of COVID-19 (the coronavirus) will cause a public health crisis in the United States?

-Very likely

-Moderately likely

-Neither likely nor unlikely

-Moderately unlikely

-Very unlikely

How concerned are you that you will contract COVID-19 by attending events on campus?

-Very likely

-Moderately likely

-Neither likely nor unlikely

-Moderately unlikely

-Very unlikely

How did you hear about this study?

-In person recruitment

-Advertisement flyers/emails

-Recommended by course instructors

-Heard from friends with sign-up in ahead

-Heard from friends without sign-up in ahead

-Come with friends on the same day

-Others

**Figure S2 Appendix. Information and experimental treatments**

Prior to undertaking a series of choices among different lunch options, participants saw two information treatments and were quizzed on the contents of the information. Participants were randomly assigned to one of two options for each of the two information treatments as described below.

**First Informational Treatment: Food Waste vs Screen Time**

(participants saw both the graphic and the bullet points)

Food Waste (FW):

- Food waste in the United States is excessive and expensive.
- In the United States, an estimated 40 percent of all food produced is wasted.
- Uneaten food at retailers, restaurants, and homes costs $161 billion annually.
-
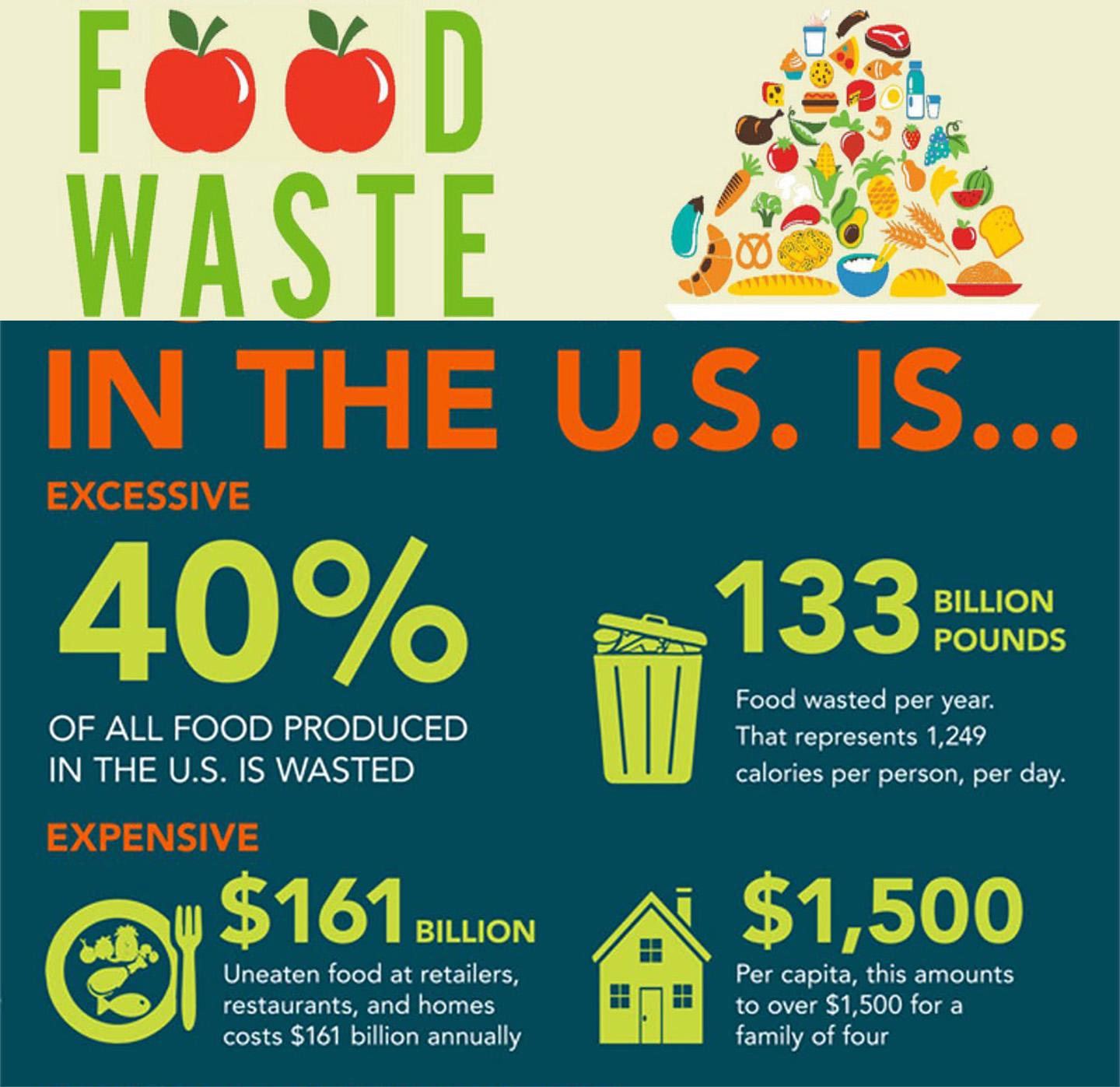


Screen Time (Screen):

- Too much screen time has been shown to have negative impacts on kids.
- More than 2 hours of screen time is too much for children over 2 years old.
- The amount of screen time children under 2 years old should have is 0 hours.


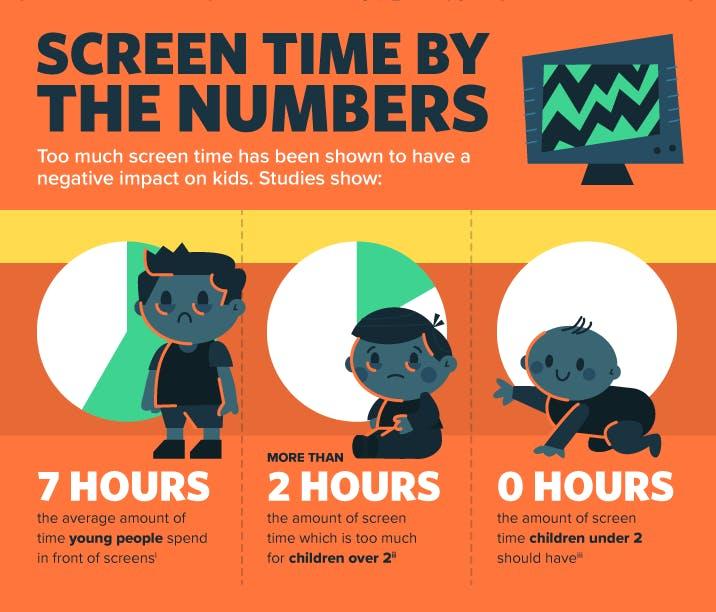


**Second Info Treatment: Nutrition vs Financial Literacy**

Nutrition:

- 39.8% of U.S. adults were obese and that another 31.8% were overweight in 2015-2016
- Nutrition experts suggest eating 500 to 700 calories for lunch.
- The healthy eating plate suggests to fill:
  - **½** of your plate with **vegetables and fruits**
  - **¼** of your plate with**grains**
  - **¼** of your plate with **healthy protein**


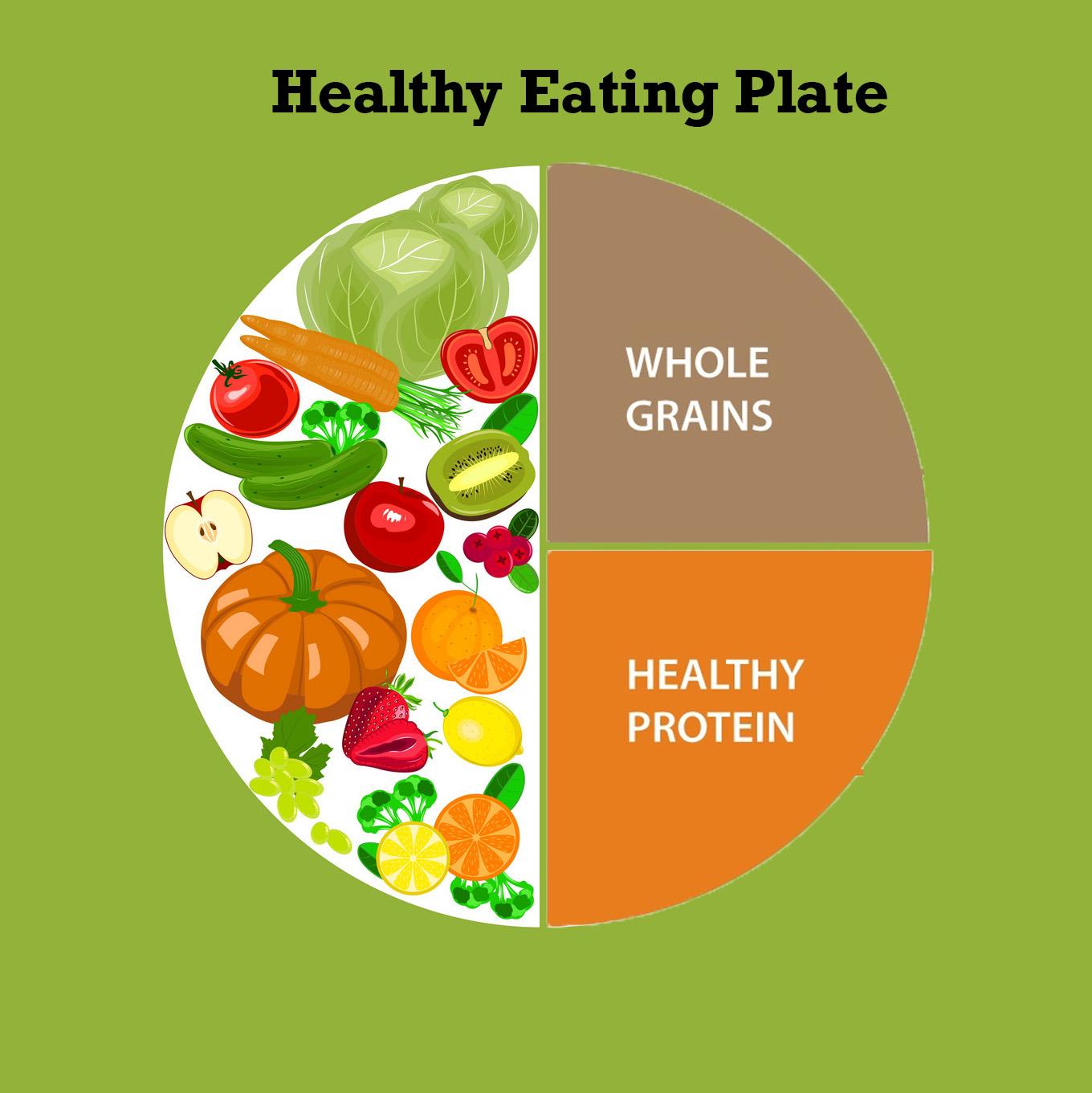


Financial Literacy:

- Half of Americans do not maintain a budget.
- One in nine people ages 18-24 uses more than 40% of his or her income to pay off debt.
- High school students who received personal financial education have higher savings and are more likely to pay debt on time.


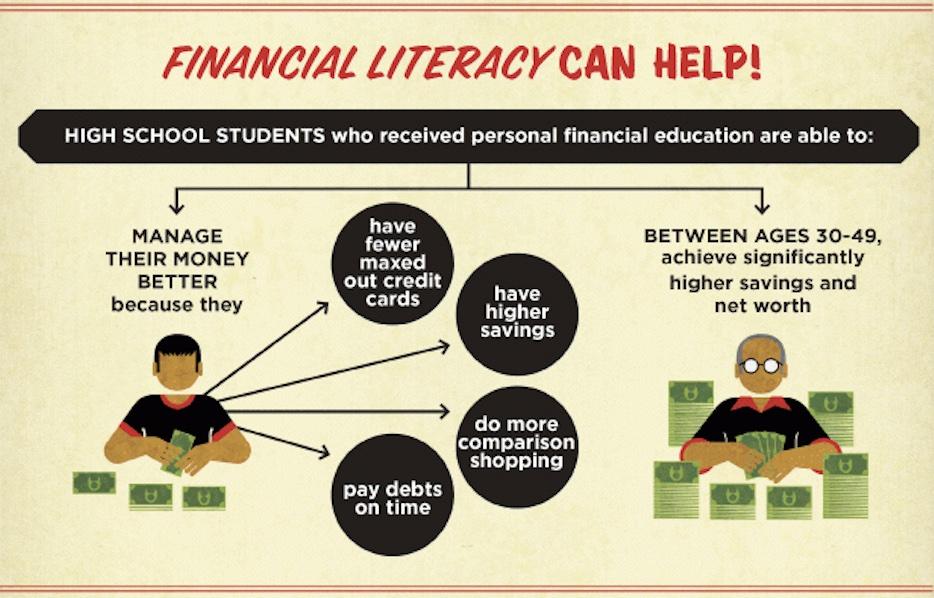


**Experimental Treatments**

The meals that were available to respondents in a particular session were randomly assigned to have the following features:

Vegetable Group:

= 1 if half of the plate served to participants contains vegetables with ¼ of the plate as mini beef meatballs

=0 if ¼ of the plate served to participants contains vegetables with half of the plate as mini beef meatballs

Large Plate:

=1 if food is delivered on a large plate

=0 if food is delivered on a small plate

Compostable Plate:

=1 if food is delivered on a compostable paper plate

=0 if food is delivered on a plastic plate

Veg Top of Menu:

=1 if the vegetable is listed on the top of the offering descriptions in menu

=0 if the vegetable is listed elsewhere on the offering descriptions in menu

**Figure S3 Classification Tree explaining Local Vulnerability**

*Note*: The bottom row features a bar graph of the proportion in that branch that were very or moderately concerned about Local Vulnerability and the number of participants in that branch. For example, 19 participants (N=19) identified as a race other than white and reported not recycling, and the proportion of this group reported being very or moderately concerned about contracting COVID-19 from attending campus events was 0.32 (height of the bar).

**Figure S4 Classification Tree explaining National Likelihood, Not Local Vulnerability**

*Note*: See Fig S3 for interpretive notes.
